# Low follow-up rate after positive prostate cancer screening in southern China: a community-based cross-sectional survey with a prospective component

**DOI:** 10.3389/fpubh.2026.1844078

**Published:** 2026-05-08

**Authors:** Zi-Qiang Li, Jin-Rong Li, Yue Li, Yue-Fen Liang, Jia-Wen Bi, Wei Li

**Affiliations:** Department of Urology, Guangxi Academy of Medical Sciences and the People’s Hospital of Guangxi Zhuang Autonomous Region, Nanning, China

**Keywords:** China, community-based, disease knowledge, family awareness, follow-up rate, lower urinary tract symptoms, prostate cancer screening, PSA

## Abstract

**Objective:**

Prostate cancer screening enables early detection of asymptomatic cases, but its public health impact depends on timely diagnostic evaluation after a positive result. However, follow-up rates after abnormal screening in community settings remain poorly characterized, particularly in underserved regions of China. This study aimed to investigate the follow-up rate and identify its determinants among men with positive prostate cancer screening results in a community-based cohort in Nanning, Guangxi, to inform targeted interventions.

**Design:**

Community-based cross-sectional survey with a 3-month prospective component.

**Methods:**

A cross-sectional study was conducted in a community in Nanning, Guangxi Zhuang Autonomous Region, from June to August 2021. Male residents aged ≥50 years (or ≥45 years with a family history of prostate cancer) who screened positive for prostate cancer (PSA > 4 μg/L) for the first time were eligible. Sociodemographic characteristics, clinical data, and psychosocial factors were collected through community health records and telephone interviews conducted 3 months after result notification. Follow-up was defined as attendance at a hospital for further evaluation within 3 months of receiving the screening result. Multivariate logistic regression was performed to identify factors associated with follow-up attendance.

**Results:**

A total of 2,939 participants underwent screening, of whom 278 (9.46%) tested positive. After excluding three cases with incomplete data, 275 men were included. Among them, 185 (67.3%) had PSA levels between 4–10 μg/L, and 90 (32.7%) had PSA > 10 μg/L. Only 81 participants (29.45%) attended a follow-up visit within 3 months. Multivariate logistic regression revealed that family awareness of the screening result (OR = 4.87, 95%CI: 1.81–13.10), presence of moderate-to-severe lower urinary tract symptoms (OR = 4.11, 95% CI: 2.14–7.89), and higher prostate cancer knowledge level (OR = 3.36, 95% CI: 1.78–6.36) were independently associated with increased odds of follow-up attendance (all *p* < 0.05).

**Conclusion:**

The follow-up rate among men with positive prostate cancer screening results in this community-based Chinese cohort was alarmingly low (29.45%). Family awareness, lower urinary tract symptom severity, and disease-specific knowledge were identified as independent determinants of follow-up behavior. These findings highlight critical gaps in the screening-to-diagnosis continuum and underscore the need for targeted interventions—particularly those enhancing family involvement, improving symptom recognition, and increasing disease knowledge—to improve follow-up rates and ultimately optimize prostate cancer outcomes in community-based screening programs.

## 1 Introduction

Prostate cancer is the most common genitourinary malignancy in men worldwide. In China, its incidence has risen sharply, from 6.72 per 100,000 in 2016 to 9.7 per 100,000 in 2022. The median age at diagnosis is 72 years, with a peak incidence between 75 and 79 years (1). As life expectancy increases and lifestyles evolve, this disease burden is projected to intensify further. Survival outcomes are strongly stage-dependent: patients diagnosed with localized disease have substantially better prognoses than those diagnosed at advanced stages (2). However, prostate cancer is often asymptomatic in its early phases, meaning that by the time symptoms emerge, the disease has frequently already progressed. In China, the proportion of patients diagnosed at advanced stages is higher than in Western countries, and the mortality-to-incidence ratio remains elevated (3).

Prostate-specific antigen (PSA) screening enables the early detection of asymptomatic prostate cancer. Chinese guidelines recommend screening for men aged 50 years and older, or from age 45 for those with a family history, and evidence indicates that higher screening frequency is associated with reduced prostate cancer mortality (4).

Nevertheless, the public health benefit of screening depends not only on coverage but also on timely diagnostic follow-up after a positive result. In the United States, follow-up rates among men with abnormal PSA findings range from 60 to 80%; in China, however, reported rates are as low as 25.5 to 31.2% (5). This low adherence represents a critical gap in the screening-to-diagnosis continuum, as delays may allow potentially curable cancers to progress. Follow-up behavior is likely influenced by multiple factors, including socioeconomic status, disease knowledge, health beliefs, social support, and the presence or absence of urinary symptoms, which may shape the perceived need for further evaluation (6). Understanding these determinants is essential for designing targeted interventions to improve follow-up adherence.

The Guangxi Zhuang Autonomous Region, an economically developing area in southern China, lacks sufficient data on follow-up rates and their determinants among community-dwelling men with positive prostate cancer screening results in urban settings. To address this gap, a community-based cross-sectional survey with a 3-month prospective follow-up component was conducted in Nanning, Guangxi, to investigate follow-up rates and identify factors associated with follow-up attendance, thereby providing evidence to inform strategies for improving prostate cancer outcomes in similar underserved populations.

## 2 Materials and methods

### 2.1 Study participants

The study comprised a cross-sectional screening phase and a 3-month prospective follow-up phase for participants with positive PSA results. Between June and August 2021, a convenience sampling method was employed to recruit participants from a community in Nanning, Guangxi Zhuang Autonomous Region. A total of 2,939 male residents who met the community prostate cancer screening requirements underwent PSA screening during the study period and were initially enrolled. Inclusion criteria were as follows: (1) met community prostate cancer screening requirements, specifically male residents aged ≥50 years, or aged ≥45 years with a family history of prostate cancer; (2) underwent PSA screening during the study period; (3) conscious and able to communicate effectively; and (4) voluntarily participated and provided written informed consent. Exclusion criteria included: (1) previously diagnosed with prostate cancer; (2) had acute prostatitis, acute urinary retention, or clinical procedures or behaviors stimulating the prostate (including prostate biopsy, cystoscopy, digital rectal examination, ejaculation, or transurethral surgery) within 2 weeks before screening where the interval was insufficient to exclude potential effects on PSA test results. All participants provided written informed consent prior to enrollment. The study protocol was approved by the Hospital Ethics Committee (Approval Number: KY-IIT-2021-02).

Given the convenience sampling strategy and the single urban setting, the generalizability of the findings may be limited. Participants who voluntarily attended community-based screening may possess higher health awareness than the general population, which could influence the observed follow-up rate and its correlates. This consideration is further addressed in the Limitations section.

### 2.2 Survey tools

#### 2.2.1 General information questionnaire

Demographic and clinical data were collected using a self-designed questionnaire. Items were selected based on a review of previously published studies examining correlates of cancer screening adherence, particularly in community-based settings (7, 8). The following sociodemographic characteristics were collected: age, educational level, marital status, health insurance payment method, disability status, children status, smoking history, health check-up habits, poverty status, family awareness of the condition, employment status, and surgical history. Clinical medical record data extracted included PSA values, family history, surgical history, chronic disease history, and family history of prostate cancer. As this questionnaire collected factual demographic and clinical information rather than psychometric constructs, formal validation procedures were not applicable.

#### 2.2.2 International prostate symptom score (IPSS)

The IPSS consists of seven questions related to urinary symptoms and one question concerning the bother associated with urination. Each of the seven symptom questions is scored on a scale from 0 to 5 points. The total score ranges from 0 to 35 points. Based on the total score, symptoms are classified as mild (1–7 points), moderate (8–19 points), or severe (20–35 points). The single bother question assesses the impact of urinary problems, with responses ranging from “delighted” to “terrible,” scored from 0 to 6 points (9). The Cronbach’s alpha coefficient of the IPSS scale is 0.86, and the test–retest reliability after 1 week is 0.92.

#### 2.2.3 Self-rated health status

Variable classification was performed based on responses to the question: “How would you rate your current health status?” Referring to the study by Li et al. (9), responses of “very satisfied,” “basically satisfied,” and “fair” were defined as good self-rated health, while responses of “dissatisfied” and “very dissatisfied” were defined as poor self-rated health.

#### 2.2.4 Barthel Index

The Barthel Index was employed to assess patients’ ability to perform activities of daily living. It includes 10 items: feeding, moving from bed to wheelchair, personal hygiene, toileting, bathing, walking, stair climbing, dressing, bowel control, and bladder control. The total score is 100 points. A score of 100 indicates independence, 61–99 indicates mild dependence, and ≤60 indicates moderate to severe dependence (10).

#### 2.2.5 Prostate cancer knowledge level questionnaire

This questionnaire was self-designed with reference to relevant literature (11, 12). It encompasses knowledge about prostate cancer, including clinical manifestations, risk factors, and screening methods. The questionnaire consists of 10 short-answer items. Each item is scored using a 4-point Likert scale: “unclear” (1 point), “insufficiently clear” (2 points), “relatively clear” (3 points), and “clear” (4 points), corresponding to the ability to narrate or describe none, a few, most, or all aspects of the item content. The maximum total score is 40 points. Based on the distribution of pre-test scores and a median split, a total score of ≥20 points was defined as a high level of disease knowledge, and a score below 20 points as a low level. In the pre-test, the Cronbach’s alpha coefficient for this scale was 0.850, and the content validity index (CVI) was 0.80, indicating good reliability and validity. Although the questionnaire demonstrated acceptable internal consistency (Cronbach’s *α* = 0.850) and content validity (CVI = 0.80) in a pilot sample of 30 community-dwelling men, it has not undergone external validation against an established gold-standard instrument for assessing prostate cancer knowledge. At the time of study initiation, no widely accepted, validated Chinese-language instrument specific to prostate cancer knowledge in community-dwelling older adults was available to our knowledge. Consequently, findings related to disease knowledge should be interpreted with appropriate caution, and direct comparisons with studies employing validated scales may be limited.

#### 2.2.6 Health-promoting lifestyle profile (HPLP-C)

The HPLP-C was used to evaluate patients’ health behaviors. This scale includes six dimensions: health responsibility, physical activity, self-actualization, interpersonal relations, and stress management, comprising a total of 40 items. Each item is scored on a 1 to 4-point scale. The total score ranges from 40 to 160 points. A score of ≥100 points was defined as having a healthy lifestyle, while a score of <100 points was defined as having an unhealthy lifestyle. A higher total score indicates a greater frequency of participation in health-promoting behaviors. The Cronbach’s alpha coefficient for this scale is 0.92 (13).

### 2.3 Operational definitions

Based on the marked increase in prostate cancer incidence among individuals aged 75 years and older (14), age was dichotomized into two groups: ≥75 years and <75 years. In accordance with clinical guidelines (15), a PSA level >4 μg/L was defined as abnormal. Further stratification was applied, with PSA levels between 4 μg/L and 10 μg/L classified as moderately elevated, and PSA levels >10 μg/L classified as highly elevated.

### 2.4 Data collection

Patients who met the inclusion criteria were enrolled between June and August 2021. All participants received their PSA test results within 1 week of screening. Three months after the result notification (i.e., between September and November 2021), a telephone follow-up survey was conducted. General information was extracted from community health records, while the remaining information (including psychosocial factors and follow-up behavior) was collected through standardized telephone interviews. Follow-up attendance was defined as having visited a hospital for prostate-related concerns within 3 months of receiving the screening result. This outcome was primarily ascertained through participant self-report during the telephone interview. To minimize potential reporting bias, self-reported information was cross-verified against available hospital medical records for a subset of participants who provided consent and whose records were accessible within the regional health information system. Discrepancies were resolved through a second telephone contact with the participant.

The 3-month follow-up window was selected based on clinical guideline recommendations emphasizing prompt diagnostic evaluation following an elevated PSA result. Specifically, the American Urological Association (AUA) and Society of Urologic Oncology (SUO) guideline on early detection of prostate cancer recommends that men with an elevated PSA should undergo further evaluation in a timely manner to avoid diagnostic delay (15), which may compromise the opportunity for early intervention. A 3-month interval has been used in prior community-based screening studies in China to define early adherence and represents a clinically meaningful period during which follow-up is most actionable.

The follow-up rate was calculated as the number of participants who attended such a visit divided by the total number of enrolled participants with positive screening results.

### 2.5 Quality control

- Study subjects were selected strictly according to inclusion and exclusion criteria and the sampling method.
- Uniform and objective indicators and scales were used. The researcher provided standardized training on data collection methods to three community nurses to control for research bias.
- During the telephone follow-up, the researcher and three community nurses explained the purpose, content, estimated completion time, and confidentiality of the study to respondents. After obtaining verbal consent, investigators explained each item and recorded responses.
- Follow-up results were entered after double-checking by two individuals. Any omissions or errors were promptly supplemented or corrected. Questionnaires with ≥20% missing content were excluded to ensure data completeness and accuracy.

### 2.6 Statistical analysis

Data were entered by two researchers using Epidata 3.1 to ensure accuracy. Statistical analyses were performed using SPSS version 25.0. Descriptive statistics were calculated based on data types. The Shapiro–Wilk test was used to assess the normality of continuous variables. As several variables deviated significantly from a normal distribution (*p* < 0.05), Spearman’s rank correlation was employed to examine bivariate associations between the included factors and follow-up attendance, with *p* < 0.05 considered statistically significant. For bivariate analysis, count data were presented as rates and composition ratios, and the chi-square test was performed.

Variables with *p* < 0.05 in the bivariate analysis were selected as independent variables for multivariate logistic regression. Follow-up status was used as the dependent variable (follow-up = 1, non-follow-up = 0). Prior to regression, multicollinearity diagnostics were conducted, confirming that all variance inflation factor (VIF) values were below 5 (ranging from 1.024 to 1.110), indicating no significant multicollinearity among the predictors (Table 1). Logistic regression analysis was performed using the forward stepwise regression method, with the *p*-value threshold set at 0.05. Variable assignments are detailed in Table 2.

**Table 1:** Collinearity analysis.

Variables	Collinearity statistics	
	Tolerance	VIF
Education level	0.963	1.039
Self-rated health status	0.901	1.110
Regular health check-ups	0.904	1.106
Family awareness of the condition	0.954	1.049
Lower urinary tract symptoms	0.970	1.031
Health-promoting behaviors	0.977	1.024
Disease knowledge level	0.977	1.024

**Table 2:** Assignment of each variable.

Variable	Assignment
Education level	Junior high school and below = 0; High school or above = 1
Self-rated health status	Good (high) = 1; Poor (not sufficient) = 0
Regular health check-ups	Yes = 1; No = 0
Family awareness of the condition	Yes = 1; No = 0
Lower urinary tract symptoms	Moderate to severe = 1; No or mild = 0
Health-promoting behaviors	≥100points = 1; <100points = 0
Disease knowledge level	superior = 1; lower = 0

## 3 Results

### 3.1 Prostate cancer screening results in community residents

**Figure 1:**
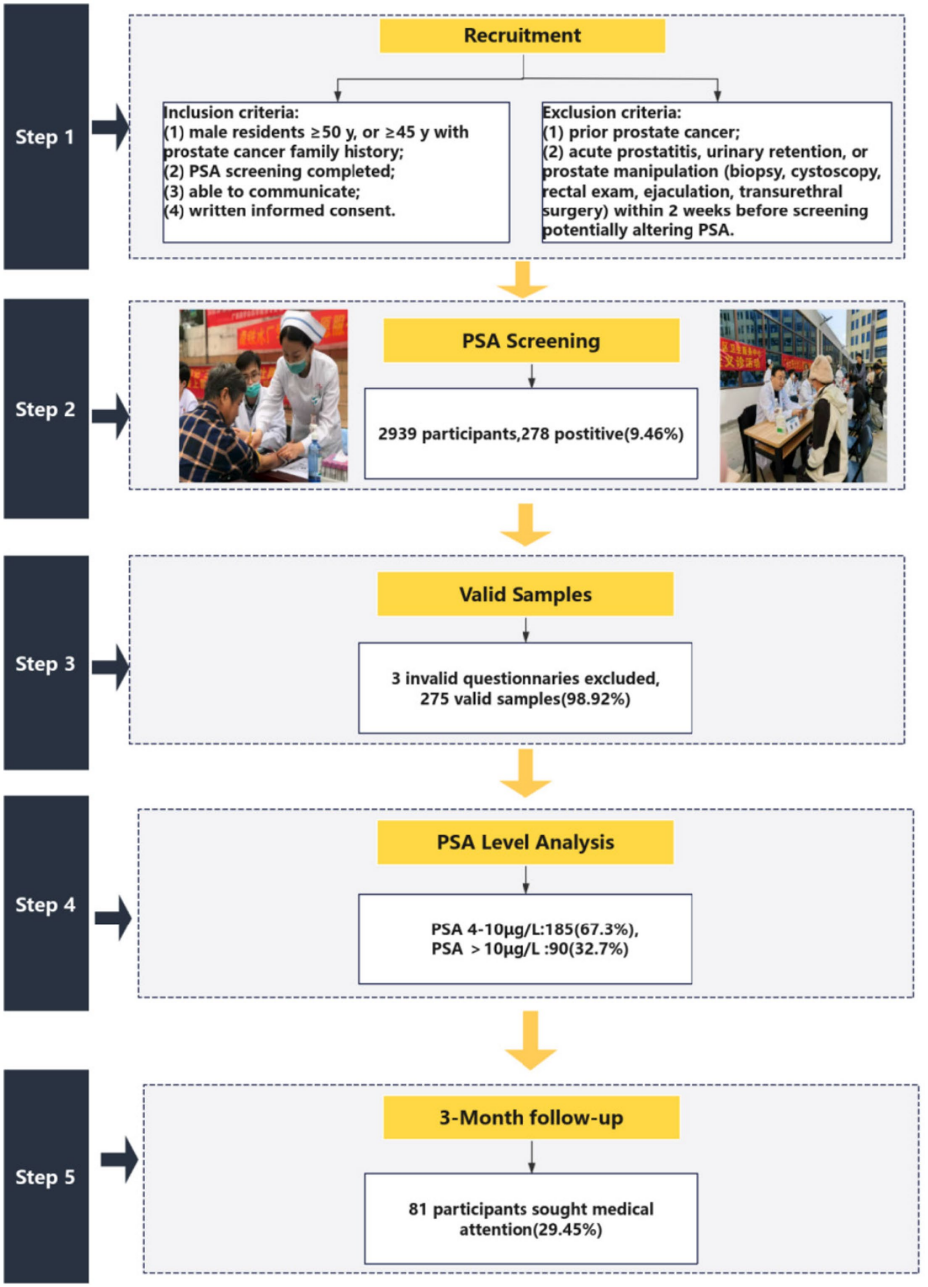
Study flow diagram.

A total of 2,939 community residents underwent prostate cancer screening. Among them, 278 individuals tested positive, yielding a positive rate of 9.46%. After excluding three questionnaires with incomplete data, 275 valid responses were retained (effective response rate: 98.92%). Of the 275 participants with positive screening results, 185 (67.3%) exhibited PSA levels between 4 μg/L and 10 μg/L, while 90 (32.7%) had PSA levels exceeding 10 μg/L. Within this cohort, 81 patients sought medical consultation within the three-month follow-up period, resulting in an overall follow-up rate of 29.45%. A participant flow diagram is shown in Figure 1.

### 3.2 Correlation analysis of factors associated with follow-up rate

Spearman correlation analysis revealed that follow-up rate was significantly correlated with several factors, including educational level (*r* = 0.123, *p* = 0.041), self-rated health status (*r* = 0.130, *p* = 0.031), regular health check-ups (*r* = 0.124, *p* = 0.040), family awareness of the condition (*r* = 0.236, *p* < 0.001), lower urinary tract symptoms (*r* = 0.277, *p* < 0.001), health-promoting behaviors (*r* = 0.130, *p* = 0.031), and disease knowledge level (*r* = 0.248, *p* < 0.001). Conversely, no statistically significant correlations were observed between follow-up rate and age, marital status, payment method, PSA level, presence of chronic diseases, history of malignancy, having children, or self-care ability (all *p* > 0.05). Detailed correlation coefficients are presented in Table 3.

**Table 3:** Correlation analysis of each item with the visits.

Item	Educational level	Education level	Marital status	payment method	PSA level	Presence of chronic diseases	History of malignancy	Having children
*r*	−0.079	0.123	0.028	−0.116	0.054	0.039	−0.079	0.021
*P*	0.192	0.041	0.649	0.055	0.375	0.519	0.190	0.730

Self-rated health status	Self-care ability	Regular health check-ups	Family awareness of the condition	Lower urinary tract symptoms	Health-promoting behaviors	Disease knowledge level
0.130	−0.064	0.124	0.236	0.277	0.130	0.248
0.031	0.287	0.040	0.000	0.000	0.031	0.000

### 3.3 Bivariate analysis of follow-up rate among community residents with positive prostate cancer screening

Bivariate analysis was conducted to compare characteristics between the follow-up group (*n* = 81) and the non-follow-up group (*n* = 194). No statistically significant differences were observed between the two groups with respect to age, marital status, payment method, PSA level, presence of chronic diseases, history of malignancy, having children, or self-care ability (all *p* > 0.05; see Table 4 for detailed statistics). However, significant differences emerged in several domains. Participants in the follow-up group demonstrated a higher proportion of individuals with high school education or above (62.96% vs. 49.48%; *χ*^2^ = 4.172, *p* = 0.041), better self-rated health (83.95% vs. 71.65%; *χ*^2^ = 4.645, *p* = 0.031), higher rates of regular health check-ups (86.42% vs. 75.26%; *χ*^2^ = 4.225, *p* = 0.040), greater family awareness of their condition (93.83% vs. 72.68%; *χ*^2^ = 15.354, *p* < 0.001), more frequent moderate-to-severe lower urinary tract symptoms (60.49% vs. 38.14%; *χ*^2^ = 11.545, *p* = 0.001), higher prevalence of health-promoting behaviors (64.20% vs. 50.00%; *χ*^2^ = 4.640, *p* = 0.031), and superior disease knowledge levels (77.78% vs. 51.03%; *χ*^2^ = 16.888, *p* < 0.001). Comprehensive comparisons are detailed in Table 4.

**Table 4:** Bivariate analysis of consultation rate of community residents with positive prostate cancer screening.

Item	Follow-up group (*n* = 81)	Non-follow-up group (*n* = 194)	*χ*^2^	*p*
Age, *n* (%)			1.709	0.191
<75 years old	58 (71.60)	123 (63.40)		
≥75 years old	23 (28.40)	71 (36.60)		
Education level, *n* (%)			4.172	0.041
Junior high school and below	30 (37.04)	98 (50.52)		
High school or above	51 (62.96)	96 (49.48)		
Marital status, *n* (%)			0.209	0.648
Single/divorced/widowed	10 (12.35)	28 (14.43)		
Married	71 (87.65)	166 (85.57)		
Payment method, *n* (%)			4.061	0.255
District-level	5 (6.17)	10 (5.15)		
Directly under the city	47 (58.02)	132 (68.04)		
Commercial insurance	9 (11.11)	23 (11.86)		
Own expense	20 (24.69)	29 (14.95)		
PSA level, *n* (%)			0.794	0.373
4 ~ 10 μg/L	45 (55.56)	119 (61.34)		
>10 μg/L	36 (44.44)	75 (38.66)		
Chronic disease, *n* (%)			0.420	0.517
Yes	51 (62.96)	114 (58.76)		
No	30 (37.04)	80 (41.24)		
History of malignancy, *n* (%)			1.725	0.189
Yes	7 (8.64)	28 (14.43)		
No	74 (91.36)	166 (85.57)		
Having children, *n* (%)			0.120	0.729
Yes	78 (96.30)	185 (95.36)		
No	3 (3.70)	9 (4.64)		
Self-rated health status, *n* (%)			4.645	0.031
Good (high)	68 (83.95)	139 (71.65)		
Poor (not sufficient)	13 (16.05)	55 (28.35)		
Self-care ability, *n* (%)			1.141	0.285
Self-care	68 (83.95)	172 (88.66)		
Mild dependence	13 (16.05)	22 (11.34)		
Regular health check-ups, *n* (%)			4.225	0.040
Yes	70 (86.42)	146 (75.26)		
No	11 (13.58)	48 (24.74)		
Family awareness of the condition, *n* (%)			15.354	<0.001
Yes	76 (93.83)	141 (72.68)		
No	5 (6.17)	53 (27.32)		
Lower urinary tract symptoms, *n* (%)			11.545	0.001
Moderate to severe	49 (60.49)	74 (38.14)		
No or mild	32 (39.51)	120 (61.86)		
Health-promoting behaviors, *n* (%)			4.640	0.031
≥100 points	52 (64.20)	97 (50.00)		
<100 points	29 (35.80)	97 (50.00)		
Disease knowledge level, *n* (%)			16.888	<0.001
Superior	63 (77.78)	99 (51.03)		
Lower	18 (22.22)	95 (48.97)		

### 3.4 Logistic regression analysis of factors associated with follow-up attendance

To further examine factors associated with follow-up attendance, multivariate logistic regression analysis was performed with follow-up status as the dependent variable (follow-up = 1, non-follow-up = 0). Independent variables included those with *p* < 0.05 in the bivariate analysis. Prior to regression, multicollinearity diagnostics were conducted, confirming that all variance inflation factor (VIF) values were below 5 (ranging from 1.024 to 1.110), indicating no significant multicollinearity among the predictors (Table 1). Variable assignments are detailed in Table 2.

The logistic regression results revealed that family awareness of the condition (OR = 4.87, 95% CI: 1.81–13.10, *p* = 0.002), presence of moderate-to-severe lower urinary tract symptoms (OR = 4.11, 95% CI: 2.14–7.89, *p* < 0.001), and higher disease knowledge level (OR = 3.36, 95% CI: 1.78–6.36, *p* < 0.001) were independently and positively associated with follow-up behavior. Detailed odds ratios, 95% confidence intervals, and corresponding *p*-values are presented in Table 5.

**Table 5:** Logistic regression analysis of consultation rate of community residents with positive prostate cancer screening.

Variable	*B*	Standard error	Wald statistic	*p-*value	OR (95%CI)
Family awareness of the condition	1.582	0.505	9.804	0.002	4.866 (1.807–13.103)
Lower urinary tract symptoms	1.413	0.333	18.050	<0.001	4.109 (2.141–7.887)
Disease knowledge level	1.212	0.325	13.907	<0.001	3.361 (1.777–6.355)

## 4 Discussion

### 4.1 Low follow-up rate among community residents with positive prostate cancer screening results

This study included 275 patients with positive screening results, of whom only 81 (29.45%) sought medical consultation within 3 months. This rate is substantially lower than that reported in studies from the United States (60–80%) and approximates those observed in developing countries (30–50%), consistent with similar domestic studies (16). These findings indicate that the follow-up rate among community residents with positive prostate cancer screening results remains concerningly low. Such low adherence diminishes the potential benefits of early cancer screening by reducing the detection of early-stage malignancies. Survival outcomes for prostate cancer patients are predominantly determined by disease stage at diagnosis, with early detection substantially improving survival rates. Although treatment modalities for prostate cancer have advanced continuously, metastatic prostate cancer remains incurable. It has been reported that the 23-year cancer-specific survival rate for patients with localized low-to-intermediate risk disease following radical prostatectomy reaches 80.4%, while the 7-year cancer-specific survival rate for patients with localized high-risk disease after radical prostatectomy is 93.0% (5). Therefore, improving follow-up rates among patients with positive prostate cancer screening results represents an urgent priority.

Existing strategies to enhance follow-up rates in early cancer screening include patient education, focused management of high-risk populations, acceleration of information technology infrastructure, and expansion of individual-level platform access (17). However, studies specifically addressing interventions for patients with positive initial screening results remain limited. Healthcare providers should strengthen management of patients with positive initial prostate cancer screening results, enhance patients’ understanding of prostate cancer, and optimize consultation processes to improve follow-up rates. In the present study, participants with informed family members, symptomatic urinary conditions, and better disease understanding were more likely to seek medical follow-up after a positive prostate cancer screening result.

### 4.2 Low disease knowledge level as a barrier to follow-up

Our findings revealed that patients with lower disease knowledge levels exhibited significantly lower follow-up rates, with low disease knowledge serving as a barrier to health-seeking behaviors—consistent with findings from other cancer screening studies (18, 19). Patients with limited disease knowledge often possess inadequate understanding of the condition, which may be associated with distorted perceptions of disease risk and lower likelihood of adopting appropriate health behaviors. Patient education is widely regarded as an important factor in supporting screening adherence and has been linked to enhanced disease knowledge levels. This necessitates not only sustained efforts by healthcare providers to improve cancer knowledge and health literacy among specific populations but also enhanced explanations addressing common concerns throughout program implementation. Health education for patients should be optimized in terms of media and methods based on the demographic characteristics of patients with positive prostate cancer screening results. Strategies may include reducing text-based education materials and increasing face-to-face interactions, voice-based communications, video content, and other age-appropriate educational modalities; shortening educational content to prevent fatigue among older adult populations; and, given that the prostate is a relatively private organ in males, special attention should be paid to protecting patient privacy.

### 4.3 Lack of family awareness as a barrier to follow-up

The study results demonstrated that patients whose families were unaware of their screening results had significantly lower follow-up rates. This finding aligns with the research conducted by Li (20), which indicated that family involvement effectively mobilizes patient motivation and improves health-related behaviors. Patients with positive prostate cancer screening results are predominantly older adult individuals who often lack awareness regarding early cancer diagnosis and treatment, and may even avoid health check-ups altogether. Family members can assist patients in correctly identifying symptoms through health education, such as distinguishing between prostate-related conditions and normal aging processes. Family accompaniment may help alleviate patients’ fear of diagnostic and treatment procedures and is associated with greater perceived security regarding healthcare seeking. Furthermore, family members serve as health supervisors who, through reminders and accompaniment to medical appointments, transform individual health responsibilities into shared family goals, thereby reducing procrastination behaviors (21, 22). Notably, the observed association between family awareness and follow-up adherence extends beyond findings from the active surveillance literature. This aligns with a recent retrospective cohort study of men with low-risk prostate cancer managed with active surveillance by Baba et al. (23), which reported that patient compliance with monitoring protocols was significantly influenced by social support and symptom perception. This convergence suggests that the behavioral determinants identified in the post-screening context—particularly family engagement and symptom awareness—may extend across the prostate cancer care continuum. Interventions designed to enhance family involvement may therefore benefit patients not only at the initial follow-up stage but also during long-term surveillance.

### 4.4 Mild or absent lower urinary tract symptoms as a barrier to follow-up

The study findings indicated that patients with mild or no lower urinary tract symptoms exhibited significantly lower follow-up rates. This observation can be interpreted through the lens of the Health Belief Model: asymptomatic individuals lack the “cue to action” provided by physical discomfort, and they may perceive lower personal susceptibility or disease severity, reducing their motivation to seek follow-up (24, 25). Mild urinary symptoms have limited impact on patients’ quality of life, and some individuals perceive urinary abnormalities as normal manifestations of aging, thereby failing to recognize their significance (26, 27). Some patients may even conceal these symptoms due to the perception that urination constitutes a private physiological behavior (28). It is possible that when lower urinary tract symptoms worsen and are perceived as a clear threat to quality of life, patients may be more inclined to reassess the etiology of their symptoms and ultimately seek medical consultation (29, 30). These findings remind healthcare providers to pay particular attention to patients with abnormal screening results but mild or absent lower urinary tract symptoms, and to strengthen health education and behavioral interventions for this population.

### 4.5 Limitations

This study has several limitations that should be acknowledged. First, participants were recruited from a single urban community in Nanning, Guangxi Zhuang Autonomous Region, using convenience sampling. This recruitment strategy introduces inherent selection bias, as individuals who voluntarily attend community-based health screenings are likely to be more health-conscious and have greater health literacy than the general population. Consequently, the absolute follow-up rate observed in this study (29.45%) may represent an overestimate relative to the broader population of screen-positive men in the region. Nevertheless, even if the true population rate were somewhat lower, the finding that fewer than one-third of screen-positive men sought timely follow-up underscores a substantial gap in the screening-to-diagnosis continuum that warrants urgent attention. Furthermore, while the absolute rate may vary across settings, the identified correlates—family awareness, symptom severity, and disease knowledge—are likely to remain relevant targets for intervention in similar urban community contexts. Generalizability to rural populations within Guangxi, to other provinces in China with differing healthcare infrastructure, and to international settings requires confirmation through future studies employing probability-based sampling across diverse geographic and socioeconomic strata.

Second, the cross-sectional design of this study precludes the establishment of causal relationships between the identified factors and follow-up behavior. Specifically, the temporal sequence between disease knowledge acquisition and follow-up cannot be definitively established. Although the survey assessed knowledge level at the time of the telephone interview (approximately 3 months after result notification), it remains possible that men who chose to attend follow-up subsequently acquired greater disease knowledge through clinical encounters, rather than high baseline knowledge driving the behavior. This temporal ambiguity is inherent to the cross-sectional component of our study and further limits causal inference. Longitudinal studies with repeated measures of knowledge and behavior over time are therefore warranted to confirm these associations and clarify the directionality of decision-making following positive screening results.

Third, data were collected through telephone follow-up interviews and community health record reviews, which may be subject to recall bias. Although standardized protocols were implemented and quality control measures were strictly enforced, some degree of information bias may persist. Additionally, certain variables, such as health-promoting behaviors and disease knowledge, were self-reported and may have been influenced by social desirability bias. While follow-up attendance was cross-verified against hospital records where feasible, this verification was not complete for all participants due to variations in record availability and inter-institutional data sharing limitations. Therefore, a degree of self-report bias may still remain.

Additionally, the disease knowledge questionnaire employed in this study, while developed based on literature review and expert consultation and demonstrating acceptable internal consistency and content validity in pilot testing, has not been externally validated against a reference standard. The absence of a widely accepted, validated Chinese-language instrument for assessing prostate cancer knowledge in community settings at the time of study initiation necessitated the use of a self-developed tool. This limitation should be considered when interpreting the observed association between knowledge level and follow-up behavior. Future research should prioritize the use of externally validated measures to enhance comparability across studies and to further confirm the role of disease knowledge in post-screening adherence.

Fourth, the 3-month follow-up window, while clinically justified as the critical period for timely diagnostic resolution, may not capture individuals who sought care after a longer delay. We did not perform formal sensitivity analyses using alternative time thresholds (e.g., 6 or 12 months) due to the closure of the prospective data collection period and the absence of extended follow-up data for this cohort. Consequently, our findings reflect early follow-up adherence and may underestimate longer-term follow-up rates. Nevertheless, the 3-month interval aligns with guideline recommendations for prompt evaluation and with definitions employed in prior Chinese community screening research. The identified correlates—family awareness, symptom severity, and disease knowledge—are therefore informative for understanding the determinants of prompt care-seeking behavior, which is arguably the most actionable target for intervention in the post-screening setting. Future studies with extended observation periods and pre-planned sensitivity analyses are warranted to assess adherence trajectories over longer time horizons.

Finally, this study focused on a limited set of potential influencing factors. Other unmeasured variables, such as psychological factors (e.g., anxiety, fear) and healthcare system characteristics (e.g., accessibility, distance to hospital), may also play important roles in determining follow-up behavior and should be explored in future research.

## 5 Conclusion

The follow-up rate among community residents with positive prostate cancer screening results in this underserved urban community of southern China was alarmingly low at 29.45%. Low disease knowledge level, lack of family awareness, and mild or absent lower urinary tract symptoms were identified as factors independently associated with non-adherence. To improve this suboptimal follow-up rate, community-based screening programs should prioritize multi-component interventions targeting: (1) enhancing prostate cancer literacy through tailored educational materials and face-to-face counseling; (2) actively engaging family members in the result notification and decision-making process; (3) educating men with mild or no urinary symptoms about the clinical significance of an elevated PSA level, regardless of their symptom status. Such comprehensive approaches may ultimately improve follow-up rates and optimize prostate cancer outcomes in community-based screening programs.

## Data Availability

The original contributions presented in the study are included in the article/Supplementary material, further inquiries can be directed to the corresponding authors.
