# Supplementary material for: Low follow-up rate after positive prostate cancer screening in southern China: a community-based cross-sectional survey with a prospective component
Source: Front Public Health. 2026 May 8;14:1844078. doi: 10.3389/fpubh.2026.1844078 (PMC13193869; doi:10.3389/fpubh.2026.1844078)
Supplement: Supplementary file 1 [file Supplementary_file_1.pdf]

Supplementary File 1: Development and Content of the Prostate Cancer Knowledge Questionnaire

1. Questionnaire Development Process

The prostate cancer knowledge questionnaire was developed through a systematic multi-step process:

Step 1: Literature review and item generation. A comprehensive review of the published literature on prostate cancer knowledge assessment was conducted using PubMed, CNKI, and Wanfang Data. Existing instruments, including the Prostate Cancer Knowledge Scale and relevant items from general cancer knowledge questionnaires, were reviewed to inform content domains and item structure. An initial pool of 15 items was generated, covering three domains: clinical manifestations (4 items), risk factors (3 items), and screening methods (3 items), plus 5 supplementary items.

Step 2: Expert consultation. The initial 15-item pool was reviewed by a panel of five experts, including two urologists with over 15 years of clinical experience, one epidemiologist specializing in cancer screening, one senior nursing specialist in community health education, and one health education specialist. Experts independently rated each item on a 4-point scale for relevance (1 = not relevant, 4 = highly relevant) and clarity (1 = unclear, 4 = very clear). Items with an average relevance score below 3.0 or average clarity score below 3.0 were flagged for revision or elimination. Based on expert feedback and consensus discussion, five items were removed due to redundancy or insufficient relevance to community-dwelling older adults, resulting in a final 10-item questionnaire. The Content Validity Index (CVI) was calculated as the proportion of items rated 3 or 4 by all experts, yielding a CVI of 0.80, indicating acceptable content validity.

Step 3: Pilot testing. The 10-item questionnaire was pilot-tested with 30 community-dwelling men aged ≥50 years who were recruited from the same community as the main study but were not included in the final analytic sample. Participants completed the questionnaire via face-to-face interview with a trained research nurse to ensure comprehension and to identify any ambiguous wording. Internal consistency was assessed using Cronbach's α coefficient, which was 0.850, indicating good reliability for an exploratory research instrument. Participants were also asked to

provide feedback on item clarity and difficulty; minor wording adjustments were made to two items to improve comprehension among participants with lower educational attainment.

Step 4: Finalization. Based on pilot testing results and participant feedback, the questionnaire was finalized. The final version consisted of 10 items administered via telephone interview in the main study to maintain consistency with the overall data collection protocol.

2. Questionnaire Items (English Translation and Original Chinese)

No.	Item (English Translation)	Item (Original Chinese)	Domain
1	Do you know what the prostate is and where it is located in the body?	您是否知道前列腺是什么，它在身体的哪个位置？	Clinical Manifestations
2	Do you know the common symptoms of prostate cancer (e.g., difficulty urinating, frequent urination, blood in urine)?	您是否知道前列腺癌的常见症状（如排尿困难、尿频、血尿等）？	Clinical Manifestations
3	Do you know that early-stage prostate cancer may have no obvious symptoms?	您是否知道早期前列腺癌可能没有任何明显症状？	Clinical Manifestations
4	Do you know the difference between benign prostatic hyperplasia and prostate cancer?	您是否知道前列腺增生和前列腺癌的区别？	Clinical Manifestations
5	Do you know the main risk factors for prostate cancer (e.g., older age, family history, high-fat diet)?	您是否知道前列腺癌的主要危险因素（如高龄、家族史、高脂饮食等）？	Risk Factors
6	Do you know that having a father or brother with prostate cancer increases your own risk?	您是否知道父亲或兄弟患有前列腺癌会增加您自身的患病风险？	Risk Factors
7	Do you know what a PSA (prostate-specific antigen) test is?	您是否知道什么是 PSA（前列腺特异性抗原）检	Screening Methods

No.	Item (English Translation)	Item (Original Chinese)	Domain
		测？	
8	Do you know that an elevated PSA level does not necessarily mean you have prostate cancer?	您是否知道 PSA 水平升高并不一定意味着患有前列腺癌？	Screening Methods
9	Do you know what further tests are recommended after an elevated PSA result?	您是否知道 PSA 升高后建议进一步做哪些检查？	Screening Methods
10	Do you know the potential benefits and risks of prostate cancer screening?	您是否了解前列腺癌筛查的潜在获益和风险？	Screening Methods

3. Scoring and Classification

Each of the 10 items was scored using a 4-point Likert scale based on the participant's self-reported clarity regarding the item content: "Unclear" (1 point), "Insufficiently clear" (2 points), "Relatively clear" (3 points), and "Clear" (4 points). The total score was calculated as the sum of all 10 item scores, yielding a possible range of 10 to 40 points.

Based on the distribution of scores in the pilot sample (n = 30), a median split was applied. The median score was 20 points. Therefore, in the main analysis, a total score of ≥20 points was defined as a high level of disease knowledge, and a score below 20 points was defined as a low level of disease knowledge.

4. Limitations

This questionnaire was developed specifically for the present study and has not undergone external validation against an established gold-standard instrument for prostate cancer knowledge. At the time of study initiation, no widely accepted, validated Chinese-language instrument for assessing prostate cancer knowledge in community-dwelling older adults was available to our knowledge. Therefore, findings related to disease knowledge should be interpreted with appropriate caution. Future research should prioritize the use of externally validated measures to confirm the role of disease knowledge in post-screening follow-up behavior.
